# High-dose amikacin in the first week of all-oral rifampicin-resistant TB treatment is safe: a single-arm trial

**DOI:** 10.5588/ijtldopen.25.0120

**Published:** 2025-11-12

**Authors:** J. Snobre, J. Gasana, T. Decroo, Y. Mucyo, B.K.M. Jacobs, L. Rigouts, I.C. Martin, N. Herssens, F. Hakizayezu, J.B. Ntihumbya, A. Kilibazayire, E. de Viron, D. Runyambo, C. Ndayishimiye, C.S. Merle, C.M. Muvunyi, P. Migambi, M.G.G. Sturkenboom, B.C. de Jong, J.C.S. Ngabonziza

**Affiliations:** 1 Institute of Tropical Medicine, Antwerp, Belgium;; 2 Rwanda Biomedical Centre, Kigali, Rwanda;; 3 Institute of Tropical Medicine Antwerp, Antwerp, Belgium;; 4 The Special Programme for Research and Training in Tropical Diseases (TDR), World Health Organization, Geneva, Switzerland;; 5 Department of Clinical Pharmacy and Pharmacology, University of Groningen, University Medical Center Groningen, Groningen, the Netherlands.

**Keywords:** tuberculosis, RR-TB, adverse events, bedaquiline, all-oral regimen

## Abstract

**BACKGROUND:**

Effective rifampicin-resistant TB (RR-TB) treatment should prevent resistance development. Bedaquiline is central to all-oral regimens, but it is threatened by rising resistance, possibly due to its delayed bactericidal effect. We evaluated the safety of strengthening the 1st week of all-oral RR-TB treatment with amikacin.

**METHODS:**

In a single-arm trial, 20 RR-TB patients received two intramuscular doses of amikacin (30 mg/kg) with lidocaine on the first and fourth day of treatment. The assumption was that none of the 20 patients would experience a grade 3–4 amikacin-related adverse event, corresponding to a primary hypothesis that less than 14% of patients have a grade 3–4 adverse event related to amikacin. Nephrotoxicity, ototoxicity, and pain post-injection were monitored. Early bactericidal effect and amikacin pharmacokinetics were measured.

**RESULTS:**

None of the 20 patients experienced a grade 3–4 adverse event during the first 2 weeks of treatment (95% confidence interval: 0–0.139). No hearing loss of any grade was observed. Pain assessment post-injections was minimal. Amikacin became undetectable within a median of 11 h.

**CONCLUSION:**

The intervention with high-dose amikacin in the 1st week of all-oral regimen was safe in this small cohort. A multi-country study is justified to investigate the efficacy to prevent acquired resistance.

Effective treatment regimens for rifampicin-resistant TB (RR-TB) should be safe, short, and strong enough to prevent further resistance. When RR-TB regimens are weak, acquired resistance can develop compromising treatment options.^1,2^ All-oral RR-TB regimens, which include bedaquiline and a fluoroquinolone but without a second-line injectable drug (SLID), are associated with acquired bedaquiline resistance in about 2.3% of patients, far exceeding the 0.1% of acquired rifampicin resistance in first-line treatment.^3^ This may be due to bedaquiline-delayed bactericidal activity, reaching effective exposure only after 1 week of treatment.^4^ During the 1st week of treatment, when the bacillary load is highest, a weak regimen can select drug-resistant mutants. A potential solution is to strengthen the 1st week of treatment with a drug having resistance prevention activity, such as SLIDs.^5^ SLIDs have been downgraded in the treatment for RR-TB due to ototoxicity concerns. However, studies show that with adverse event monitoring, the risk of SLID-related ototoxicity is significantly lower than literature reports.^6^ Also, ototoxicity is linked to cumulative exposure with studies mainly reporting on patients exposed during more than 4 months in traditional RR-TB treatment.^7,8^

In this study, we added amikacin, the most potent SLID,^9^ to strengthen the 1st week of a 9- to 11-month all-oral regimen. The regimen included bedaquiline (6 months) with a fluoroquinolone, ethionamide, ethambutol, high-dose isoniazid, pyrazinamide, and clofazimine for 4 months, followed by a fluoroquinolone, clofazimine, ethambutol, and pyrazinamide for 5 months. Research from the Centers for Disease Control and Prevention, Atlanta, showed that baseline SLID resistance greatly increased the risk of acquired fluoroquinolone resistance.^10,11^ Also, in Pakistan, bedaquiline resistance was significantly more frequent in regimens without SLIDs.^12^ The intervention involved two intramuscular amikacin doses (30 mg/kg) on days 1 and 4, co-administered with lidocaine, based on pharmacokinetic and safety considerations (detailed in Methods). Given challenges in enrolling large numbers of RR-TB patients, the lengthy turnaround time from designing randomised clinical trials to results, and the need to strengthen regimens to prevent resistance, exploring adaptive trial designs is essential. Since the standard regimen for RR-TB does not include amikacin, and our primary objective was to assess amikacin-related adverse events, this pilot safety study was designed as a single-arm trial with a fixed safety threshold in Rwanda. Given the rarity of RR-TB cases in the country and the need to generate timely evidence to inform a subsequent larger multi-country study (where safety will also be a key endpoint), we planned to enrol approximately 20 patients over 1 year. Based on this constraint, we hypothesised that none of the 20 patients would experience a grade 3–4 adverse event. This assumption results in an upper bound of the one-sided 95% confidence interval (CI) of 14%, which was therefore selected as the safety threshold (see power calculation in Methods). With this sample size, we aimed to test whether the rate of serious adverse events during the first 2 weeks of treatment was 14% or lower (primary objective). Although this threshold was determined on a statistical basis, it aligns with available clinical literature. A systematic review reported a 10.2% rate (95% CI: 6.3%–16.0%) of treatment interruption due to amikacin toxicity when used for ≥4 months.^13^ However, this likely underestimates the true burden of toxicity, as some Grade 3 adverse events may have gone undetected, and other studies have documented higher rates of severe ototoxicity.^12^ Therefore, a threshold of 14% appears reasonable as a conservative upper limit for this pilot study. Secondary objectives included describing all other adverse events at the end of treatment week 2 and assessing post-injection pain.

## METHODS

This single-arm trial followed the published protocol.^14^ Patients were co-enrolled in the ShORRT (All-oral shorter treatment regimen for multidrug and RR-TB) study.^15^ All patients with RR-TB in Rwanda were screened, and 20 patients were enrolled.^14^ The intervention with amikacin and pain assessment using the Wong-Baker scale are described in the study protocol.^14^ Safety and laboratory assessments are outlined in Table 1. This article describes the safety and adverse events at 2 weeks of treatment. The final end-of-study analysis will focus on end-of-treatment outcomes, outcomes 12 months post-treatment, and acquired resistance.

**Table 1.** Schedule of assessments for the STAKE study.

	Day 0	Day 1	Day 3^**A**^ 4	Day 7	Day 14	M 1/2	M 3–9	PT 6/12
Xpert XDR	X							
AMK injection (30 mg/kg)		X	X					
Pain scale^**B**^		X	X					
Quantified sputum smear microscopy	X	X	X	X	X	X	X	X
Solid and liquid culture^**C**^	X	X	X	X	X	X	X	X
Audiometry^**D**^	X		X		X	X		
Blood sampling^**E**^		X	X					
Creatinine clearance	X		X		X			
Adverse events form^**F**^					X	X	X	X

M = month; d = days; PT = post-treatment month; AMK = amikacin.

A Audiometry and creatinine clearance (+ AMK trough level) were determined on D3 to allow time for physician interpretation and clearance before D4 AMK injection.

B Describe post-injection pain on a 0–10 pain scale (the Wong-Baker FACES pain rating scale) at 0, 15, 30, and 60 min after the injection of AMK with lidocaine, as well as the next morning.

C Culture: time to positive culture and number of culture forming units on days 1 (first day of RR-TB treatment, sample collected before the first dose), 4 (sample collected just before the second dose), 7, and 14. For monthly culture, as per routine monitoring, only data on positivity (positive, negative, contaminated, not done) were collected.

D Audiometry: at baseline (day 0, before start of treatment), day 3 (before second administration of AMK), day 14, M1, M2 (as ototoxicity may emerge with delay since administration), and any time thereafter in case of hearing disturbances.

E The concentrations of AMK: just before administration of AMK (concentration of drugs other than AMK) and 2 and 6 h after the administration of AMK, when the first (day 1) and second dose (day 4) of AMK are administered.

F The table only included the schedule of cumulative adverse event report forms. However, throughout directly observed therapy, patients were monitored for any potential adverse event actively and by passive reporting.

### Inclusion and exclusion criteria

The study included patients aged 18–64 with bacteriologically or molecularly confirmed TB with evidence of resistance to at least rifampicin (ShORRT), who gave written informed consent. The molecular Xpert Ultra test was repeated for each patient upon their arrival at the clinic. Only patients with confirmed evidence of RR-TB were included in the study. Exclusion criteria included 1) any abnormality (>20 dB average hearing loss) on baseline audiometry, using the average hearing loss at frequencies of 500, 1,000, 2,000, and 4,000 Hz, 2) a history of kidney disease or baseline estimated glomerular filtration rate (eGFR) below or equal to 60 mL/min/1.73 m^2^, 3) pregnant or breastfeeding women, 4) a history of previous injectable-drug-based TB treatment, 5) documented resistance to SLIDs, or 6) use of non-steroidal anti-inflammatory drugs or diuretics.

### Study design rationale and power calculation

To generate timely safety data for a planned multi-country study, we designed this pilot to enrol 20 patients over 1 year in Rwanda. We hypothesised that none of these 20 patients would experience a grade 3–4 adverse event related to the use of amikacin. This assumption results in an upper bound of the one-sided 95% CI of 14%

For the primary analysis, we calculated the rate and the one-sided 95% CI to assess whether the upper 95% CI bound was below 14%.

• Null hypothesis: 14% of patients experience a grade 3–4 adverse event related to amikacin.
• Alternative hypothesis: less than 14% of patients experience a grade 3–4 adverse event related to amikacin.
• *P* value for a binominal test with *P*0 = 0.14 (*P* = 0.14 under the null hypothesis) for 0 events is (1 − 0.14) 20 = 0.049, that is, <5%.
• In case the probability of having a grade 3–4 adverse event likely or definitive related to the use of amikacin would be 1%, then the chance of having 0 of 20 patients with this safety endpoint is 81.8% ([1 − 0.01] 20 = 0.818 = 81.8%) providing ∼80% power to detect this safety endpoint at a probability less than 1%.

In summary, with 20 patients, we had ≥80% power to reject the null hypothesis of *P*0 = 0.14 in favour of the alternative of *P* < 0.14 assuming that *P* ≤ 0.01.

### Laboratory procedures

Xpert MTB/RIF Ultra and MTB/XDR were used for confirmation of rifampicin resistance and exclusion of SLID resistance. Routine testing included acid-fast bacilli Ziehl-Neelsen microscopy, solid/liquid culture, colony forming unit (CFU) counts, and determination of amikacin serum levels. Detailed procedures are provided in the study protocol.^14^

### Rationale for the intervention of two high doses of amikacin

The 30 mg/kg dose was based on pharmacokinetic and safety considerations. Amikacin’s bactericidal effect is maximised at a Cmax/MIC ratio of at least 10 at the site of infection^5^ translating to a serum Cmax/MIC ratio of 75 considering poor penetration of SLIDs in lungs.^5^ Amikacin’s toxicity is linked to cumulative exposure, and two doses of 30 mg/kg amikacin represent only a small fraction of traditional RR-TB treatment exposure. Indeed, amikacin has been found safe and effective for the treatment of TB at 25 mg/kg in 3 weekly doses.^16^ Finally, aminoglycosides show prolonged post-antibiotic effect, supporting intermittent dosing.

### Amikacin exposure

Venous blood was drawn 2 and 6 h after injections on days 1 and 4. A trough concentration was drawn on day 3. Serum samples were stored at −20°C until analysis using a validated immuno-assay technique on an Architect C8000 (Abbott) at the University Medical Center Groningen. Maximum concentrations at 1 h after intramuscular injection (C_1_) and cumulative area under the concentration-time curve (AUC_cum_) were estimated using a one-compartmental population pharmacokinetic model of amikacin and the Batchfit module of Edsim++, version 2.50 (Mediware, Prague, Czech Republic, Table 5).^17^ The intramuscular absorption constant (k_a_) was set at 3.0 ± 3.0/h.^18^

### Safety assessment, data management, and monitoring

Safety assessment, data management, and monitoring were performed according to the study protocol (Table 1). Briefly, patients were admitted at the clinic and systematically monitored for adverse events graded using the ‘Common Terminology Criteria for Adverse Events’.^14^ Data were entered into REDCap, with quality monitoring by the Clinical Trial Unit at the Institute of Tropical Medicine in Antwerp and the Rwanda Food and Drug Authority.

### Statistical methods for primary and secondary outcomes

For the primary analysis, we calculated the one-sided 95% CI for the primary safety endpoint and assessed whether the upper bound was below 14%. For microbiological results, we used a linear mixed-effects model to assess the association between culture time to positivity (TTP) and time since treatment initiation. For semi-quantitative microscopy results, we utilised a cumulative logistic random-effects model to test the overall trend of smear positivity over time. We applied Wilcoxon signed-rank test to compare results between different time points. CFU count analysis was excluded due to contamination in 34% of samples, falling below the 70% protocol-defined threshold. All other analyses were descriptive.

### Ethical statement

Details about confidentiality and ethics are described in the study protocol.^14^ The study follows European Union’s General Data Protection Regulation, and approval was obtained from the Ethics Committee of Rwanda (N459/RNEC/2022) and Rwanda Food and Drug Authority (DIS/FMT/050), University Hospital Antwerp, Belgium (No. 3330 - Edge n/a - BUN B3002022000108 of 08/08/22) and the Institute of Tropical Medicine (ITM) Internal Review Board (No 1567/22 of 14/06/22). Written informed consent was obtained.

## RESULTS

Seventy-five patients were screened, with 55 excluded and 20 pulmonary TB patients enrolled between 1 March 2023 and 16 January 2024 (Figure). All patients received the injections and were followed up to day 14, with complete information for the primary objective. Most patients were newly diagnosed (15, 75%); five had prior first-line treatment (3 relapse, 2 failure) (Table 2). Xpert XDR at baseline showed resistance to isoniazid in 18 patients and to ethionamide in four. None had resistance to SLIDs or fluoroquinolones.

**Figure.**
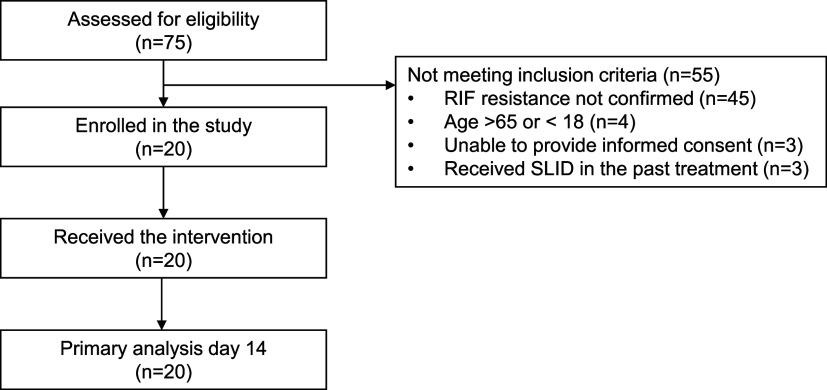
Flowchart patients enrolled in the STAKE study. RIF: rifampicin; SLID: second-line injectable drug.

**Table 2.** Patient characteristics at enrolment.

Characteristic	Median (range)
Age (y)	38.5 (27–62)
Sex male (n, %)	15 (75)
Bodyweight (kg)	51.3 (37.0–63.0)
BMI (kg/m^2^)	18.1 (13.1–22.4)
BSA (m^2^)	1.60 (1.31–1.80)
Administered dose of amikacin (mg)	1,500 (1,000–1,750)
Administered dose of amikacin (mg/kg)	28.4 (27.0–31.3)

Values are described as median (range) unless otherwise specified.

BMI = body mass index; BSA = body surface area.

Thirteen participants were HIV-negative, four were HIV-positive (all on ART), and three had an unknown status. None presented neuropathy or history of kidney disease.

Common symptoms included cough (19, 95%), night sweats (13, 65%), fever (12, 60%), chest pain (5, 25%), and haemoptysis (4, 20%). Lung abnormalities (19, 95%) such as lung crackles and decreased air entry were common. All had chest X-ray cavities at screening.

### Primary analysis

The hypothesis that fewer than 14% of patients treated with amikacin would have grade 3–4 adverse events at day 14 after 2 weeks of treatment was confirmed. Among the 20 patients, no such events were observed (95% CI of 0–0.139, *P* = 0.049).

### Secondary objectives

Ototoxicity assessments via audiograms showed no amikacin-related adverse events with average hearing loss and single-frequency increase on days 4 and 14 remaining below the grade 1 threshold adverse event (20 dB) from baseline (Table 3). For nephrotoxicity, one individual experienced a transient eGFR reduction from 114 to 89 mL/min/1.73 m^2^ on day 4 (grade 1 adverse event), normalised by day 14. All others maintained a GFR above 90 mL/min. A single case of mild epigastric pain (grade 1 adverse event) unrelated to amikacin resolved with antacids.

**Table 3.** Audiometric, renal function, and pain assessments in the first 2 weeks of the STAKE study.

	Day 1	Day 4	Day 7	Day 14
**Average hearing loss compared with baseline (dB)**	**Average (range) for 20 patients**			
Left ear	10.6 (2.5–16.2)	10.0 (5–17.5)	NT	11.2 (5–18.8)
Right ear	12.5 (5–17.5)	11.2 (6.25–18.8)	NT	11.2 (7.5–18.8)
eGFR (mL/min/1.73 m^2^)	127 (105–139)	111 (89–150)	NT	128 (99–150)
**Pain post-injection**	**Median–IQR (range) for 20 patients**			
0 min	0–0.25 (0–5)	0–0 (0–4)		
15 min	0–0 (0–2)	0–0 (0–2)		
30 min	0–0 (0–2)	0–0 (0–1)		
60 min	0–0 (0–2)	0–0 (0–1)		

dB = decibel; eGFR = estimated glomerular filtration rate calculated with the CKD-EPI formula at baseline; IQR = interquartile range.

Pain assessment post-injections revealed minimal discomfort (Table 3 and Supplementary Data) with a median score of ‘0/10’ at all time points. At most 5 of 20 patients reported any pain resulting in inter-quartile ranges of approximately ‘0/10.’ The highest scores immediately after injection were ‘5/10’ on day 1 and ‘4/10’ on day 4, both from the same patient. At 15 min and any later time point, only that same patient had pain, never higher than ‘2/10.’

### Early bactericidal activity assessments

#### Time to culture positivity

TTP increased for all 20 patients from day 1 to day 7. From day 7 to day 14, 15 patients (75%) showed further increases, while 5 (25%) showed decreases. The median TTP at 2 weeks was 390 h (Table 3). A linear mixed-effects model showed a significant TTP increase of 13.2 h per day since treatment initiation (*P* = 0.0002). Wilcoxon signed-rank tests revealed significant differences (*P* < 0.05) between all time points, except day 7 versus day 14 (*P* = 0.22).

#### Smear microscopy

Two sputum smears per time point were analysed, with the highest result taken (Table 3). The evolution of microscopy results in the first 2 weeks of treatment is described in Table 3. As an exploratory analysis, we tested if the smear positivity (as an ordinal variable) was significantly associated with the time since treatment initiation. We used a cumulative logistic random-effects model to test for an overall trend and additionally tested if the smear results were significantly different between the different time points with the Wilcoxon signed-rank test (for paired samples). The overall trend was significantly decreasing (*P* value = 0.0001). All differences at two time points apart (day 1 vs. day 7, day 1 vs. day 14, and day 4 vs. day 14) were significantly different (*P* < 0.05), while those one time point apart were not.

#### Amikacin exposure

The median administered amikacin dose was 1,500 mg and 28.4 mg/kg (Table 2). One patient showed a very low amikacin concentration at 2 h (C_2_, 4.8 mg/L) and 6 h post-injection (C_6_, 2.2 mg/L) on day 1. On day 4, two patients showed a very small difference for C_6_, compared to C_2_. These four data points (C_2_ and C_6_ on day 1 for patient 1 and C_6_ on day 4 for both patients) were omitted in the pharmacokinetic analysis. The median C_1_, calculated at 1 h post-injection for days 1 and 4 combined, was 66.8 mg/L (28.0–130 mg/L), which decreased after 2 and 6 h post-injection (Table 4). All concentrations at day 3 were < limit of quantification (1.5 mg/L). The median time post-injection to reach an amikacin concentration of <1.5 mg/L was estimated at 11.2 h. The AUC_cum_ was 527 mg·h/L (306–763 mg·h/L) (Table 4). Pharmacokinetic parameters used for the analysis are detailed in Table 5.

**Table 4.** Time to culture positivity and sputum smear microscopy results over the first 2 weeks of treatment.

	Day 1	Day 4	Day 7	Day 14
Culture time to positivity (hours)	135 (77–348)	249 (141–348)	289 (85–1,078)	390 (70–669)
**Smear microscopy**	**Number of patients**			
3+	10	9	6	3
2+	3	4	5	6
1+	4	4	5	3
Scanty	3	3	3	5
Negative	0	0	1	3

Time to positivity in liquid culture (hours) is presented as median and range for each time point. Sputum smear microscopy results are shown as the number of patients per semi-quantitative category (3+, 2+, 1+, scanty, negative).

**Table 5.** Amikacin pharmacokinetics in study participants.

Parameter	Median (range)	(IQR)
C_1_ > 60 mg/L (n, %) (10)	12 (60%)	
C_1_ (mg/L)	66.8 (28.0–130)	(44.8–78.1)
C_2_ (mg/L)	48.2 (4.8–84.6)	(34.4–59.6)
C_6_ (mg/L)	13.4 (2.2–72.4)	(9.6–19.0)
Concentration at day 3 (mg/L)	<1.5 mg/L^**A**^	
AUC_cum_ (mg·h/L)	527 (306–763)	(477–582)
Estimated post injection time when amikacin concentration <1.5 mg/L (h)	11.2 (4.8–27.0)	(10.6–12.8)

Median values with ranges and interquartile ranges (IQR) are shown for pharmacokinetic parameters. C_1_, C_2_, and C_6_ represent estimated amikacin concentrations at 1, 2, and 6 h after intramuscular injection (pooled data from days 1 and 4).

AUC_cum_ = cumulative area under the time concentration curve.

A All concentrations measured on day 3 were below the limit of quantification (1.5 mg/L).

## DISCUSSION

Our pilot study indicates that the intervention with two high-dose amikacin injections with lidocaine in the 1st week of RR-TB treatment is safe and well tolerated. Additionally, it provides insights into pharmacokinetics of high-dose amikacin. None of the 20 patients experienced an amikacin-related adverse event. No hearing loss of any grade was observed. Amikacin cleared quickly with levels <1.5 mg/L within a median of 11 h. Pain post-amikacin injection mixed with lidocaine was negligible, which aligns with other studies^19,20^ supporting lidocaine co-administration as routine practice.

SLIDs have been downgraded in RR-TB treatment due to ototoxicity concerns. However, given the increasing resistance to bedaquiline and challenges in BPaL(M) implementation (lack of adequate diagnostic tests for bedaquiline and pretomanid resistance, high incidence of linezolid toxicity^21^), it is important to investigate how drugs with strong resistance-preventing activity, such as SLIDs, can be safely added. Larger studies should study whether a relatively low cumulative dose of amikacin can strengthen the early phases of bedaquiline-containing regimens. While the primary endpoint of this study was safety, we also collected data on the early bactericidal activity as a proxy for efficacy. We found a median TTP of 390 h at 2 weeks of treatment. Further studies are needed to compare the early bactericidal activity of recently introduced BPaL(M) regimen with and without amikacin injections.

Twelve patients (60%) reached C_1_ > 60 mg/L, a concentration that has been proposed in a recent review on high-dose amikacin.^22^ This 60% is comparable to the 60%–82% of patients that showed C_1_ > 60 mg/L 30 min after a 30-min intravenous infusion of 25–30 mg/kg amikacin.^22^ The AUC_cum_ was <1% of the AUC_cum_ of 87,000 mg·h/L that is associated with a 10% probability of ototoxity.^5^

Our study has some strengths. Patients diagnosed with RR-TB nationwide were evaluated for enrolment, increasing the generalisability to the Rwandan context. Generalisability to other countries may be limited by differences in genetic susceptibility to aminoglycoside-induced ototoxicity, patients’ nutritional status, comorbidity profiles, and health care delivery systems. Additionally, when evaluating the efficacy of the intervention in preventing acquired drug resistance, it will be important to consider differing baseline resistance profiles, such as the prevalence of fluoroquinolone resistance, which is notably low in Rwanda compared to many high-burden countries as well as previous exposure to SLIDs.

Our study is integrated into the larger ShORRT study that assesses the effectiveness of the 9-month regimen with bedaquiline without amikacin, with follow-up extending to 1 year post-treatment. The study also has some limitations. While it offers insights into the potential early bactericidal activity of this regimen using TTP and semi-quantitative smear microscopy, the absence of a control arm limits the evaluation of the specific contribution of amikacin. These proxy endpoints with newly acquired resistance need to be validated in larger cohorts powered to study acquired resistance.

## CONCLUSION

The intervention with two high-dose intramuscular injections of amikacin in the 1st week of bedaquiline-containing RR-TB treatment is safe and well tolerated. End-of-treatment outcomes will be described in the final end-of-study analysis. Effectiveness in preventing drug resistance needs to be evaluated through multi-country studies, also with newer regimens such as BPaL(M).

## Supplementary Material


