# Supplementary material for: High-dose amikacin in the first week of all-oral rifampicin-resistant TB treatment is safe: a single-arm trial
Source: IJTLD Open. 2025 Nov 12;2(11):655–61. doi: 10.5588/ijtldopen.25.0120 (PMC12617081; doi:10.5588/ijtldopen.25.0120)
Supplement: Supplementary file 1 [file ijtldopen25-0120_supplementarydata1.pdf]

Table S1 Pain rates reported in the STAKE study

record_id	postinjection_day_1	15min_day_1	30min_day_1	60min_day_1	postinjection_day_4	15min_day_4	30min_day_4	60min_day_4
2023-01-016	3	0	0	0	0	0	0	0
2023-01-019	1	0	0	0	1	0	0	0
2023-01-026	0	0	0	0	0	0	0	0
2023-01-043	0	0	0	0	0	0	0	0
2023-01-044	0	0	0	0	0	0	0	0
2023-01-046	2	0	0	0	0	0	0	0
2023-01-048	0	0	0	0	0	0	0	0
2023-01-057	0	0	0	0	0	0	0	0
2023-01-058	0	0	0	0	0	0	0	0
2023-01-060	0	0	0	0	0	0	0	0
2023-01-061	0	0	0	0	0	0	0	0
2023-01-063	0	0	0	0	0	0	0	0
2023-01-065	0	0	0	0	0	0	0	0
2023-01-071	1	0	0	0	0	0	0	0
2023-01-073	0	0	0	0	0	0	0	0
2023-01-075	0	0	0	0	0	0	0	0
2023-01-083	0	0	0	0	0	0	0	0
2023-01-085	5	2	2	2	4	2	1	1
2024-01-001	0	0	0	0	0	0	0	0
2024-01-002	0	0	0	0	0	0	0	0

Table S2 The population pharmacokinetic model is the one described in Dijkstra et al.
Vd volume of distribution (L/ kg lean body mass corrected). Ke renal elimination rate constant (1/h.(ml.min.1.73m2), Ka absorption constant after intramuscular injection (/h) are reported as mean ± standard deviation. Bioavailability after intramuscular injection is unknown and was therefore fixed at 1.

Pharmacokinetic parameter	Population pharmacokinetic model (Dijkstra et al, 2015)	STAKE (n=20)(mean ± standard deviation)
Vd (L/kg LBMc)	0.207 ± 0.0664	0.345 ± 0.160
K_e (1/h/(ml/min/1.73m^2))	0.00384 ± 0.00143	0.00335 ± 0.00157
Half-life (h)	Not reported	2.15 ± 1.00
K_a (/h)	3.0 ± 3.0 (Amikacin SMPC)	3.68 ± 1.80
